# The Influence of the Culinary Art upon Health

**Published:** 1855-08

**Authors:** 


We commend the following article to the careful perusal of our readers, and to that of their wives. It abounds in good sense and valuable hints. Particularly do we indorse the recommendation of roasted meats, and the objections to baked, as well as the commendation of broiling. It is satisfactory to know that the gridiron, which so preserves the flavor and juiciness of meats, is also to be recommended for chemical and dietetic reasons.—*Ed. Buff. Med. Journal.*

*The Influence of the Culinary Art upon Health.* By R. C. Mackall, M. D., of Savannah, Ga. (Published by request of the Medical Society of the State of Georgia.)

The sum of human life is not made up of great, but of small things. Man, with all his boasted likeness to the gods, presents himself to the physician chiefly as a mere animal. In infancy, the most helpless of all creatures, his manhood is too often stained by the weaknesses of the first stage, and his old age is the yesterday of childhood returned. The few who reach "the lean and slippery pantaloon" are sad satires upon the God-like. The big manly voice forever gone, the staff and tottering gait are but a mocking of the first attempts to walk from chair to nurse.

The interlude between the chair and staff is a part of the same play, in which we witness a continued struggle between strength and feebleness—physical and moral. The single aim of man is selfish. The gratification of his passions and appetites is the goal of all his labors. To this end hero and sage, artisan and serf, work. And, now, in the 19th century, the fields from which they cull their gratifications, present the same varieties to their tastes as were enjoyed by the classic, yet moral ancients. The great of the past, were only great then, as now, when the world looked on. Then, as now, poet and orator, statesman and sage, disdained not to dwell upon the little affairs of life. Troy's historian could degrade the melody of his verse to a description of the vile cookery of his countrymen, and the names Lentulus, Fabius, Cicero, assure us that even the masters of a world never forgot that they had stomachs. The fame of Cato smells of the kitchen, and Vitellius could get up a dish that a modern Frenchman would not attempt to rival.

If, then, the immortal ancients would stop to tell us how to prepare a feast —we moderns—who almost eat as well as travel by steam—may well pause and look to our laurels in the regions where Toby and mamma preside,—notwithstanding a wise man has written, "put a knife to thy throat if thou be a man given to appetite," and a wit said in the teeth of the proverb, "*qu'un cuisinier est un mortel divin.*"

Now, although I cannot agree with either the Hebrew sage or the French poet, I sincerely believe that kitchen lore is too much neglected by the ultra utilitarians of our day and country. I am thoroughly satisfied that to this neglect we are indebted for a large amount of our work, and that if we had in every house in our broad land—if not one of Voltaire's "mortel divins," a well instructed cook—we might soon lay aside many of our vile compounds, and see disappear from before an unjaundiced public eye, a hundred quack certificates in favor of liver pills, etc.

American cookery in its influence upon health, bears about the same relation to the many varied forms of dyspepsia, as malaria does to our miasmatic fevers. The poison of the one is brewed in a pot or frying pan—that of the other in a fen or marsh. The swinish feast with which Penelope's suitors were regaled may indeed be found with all its classic taste and indigestive vileness in the modern cuisine, and often so much improved that those ancient lovers themselves would hardly recognize the noble dish.

Now, although physicians are not cooks, yet, as conservators of the public health, it is their duty to understand the art of cookery in its relations to the physiology of digestion. To the almost entire neglect of the kitchen, we have been for generations searching earth, air, rivers, ponds, marshes, etc., for causes of disease. This should have been done, but not to the leaving of the other undone. Unimpaired digestion would greatly assist us in all our labors, as it certainly would our unfortunate patients, even in the midst of malaria or the *absence of ozone.* And, although sound stomachs might not deprive epidemics of their subjects, they certainly would rob them of many victims; for if the prima via is in good order, the traveler has a tolerable surety against accident. Thus some of the opprobrium of medicine would be removed, and the now wide field of quackery considerably narrowed.

In fulfilling the duty imposed upon me by the society, it will be my endeavor rather to attract laborers to the Augean work of clear'ng the kitchen and enlightening cooks, than to attempt the task myself. My effort will be to give a general view of the art of preparing food—to notice some of the abuses of the art, with the results which follow them.

Addressing my observations to physicians, it is unnecessary to enter into an elaborate argument to prove that there is a near connection between the culinary art and health, or that the influence of the former upon the latter is not beneath our consideration. But, in order to exhibit this fact so as to attract special notice, it may be well first to turn the picture, holding up to your view the relations between the abuse of the art and disease.

Man being an omnivorous animal, his stomach is by nature fitted for the digestion of every thing that is good for food—indeed, for many things not food, physiologically considered. From earth, sea and rivers, he gathers the thousand articles he feeds upon. Nature has been lavish in her provisions for his wants. But although she thus amply provides for his varied taste and capricious appetite, she has not for him as for the brute, prepared her bounties for his organs or digestion. To reasoning man it is left, either to be satisfied with a few of the fruits of the earth, or to devise modes by which to render the animal and vegetable kingdoms subservient to his desires.

With the gift of superior intelligence, she has made him the custodian of his own powers, subject only to her laws, for the violation of which she holds him strictly responsible. Yet, ever generous, she has provided him with more than one sentinel to guard the approaches to the castle of his health, and in the castle itself, has placed a faithful keeper. With ever ready wit, corporal smell and sergeant taste, guard, the one the outpost, the other the entrance, whilst private nausea, even to the wall, strives to cast out every rude invader. Yet, notwithstanding nature's lavish kindness, and too often in spite of sentinels and keeper, the castle is entered successfully and its wal s sadly injured. Thus man becomes a vandal to his own stomach, invading it with reckless violence, until its integrity is destroyed, and, in revenge, it curses him with disease for the residue of his days.

But, in our own day and country, I believe, more harm is done to this vital organ through ignorance than through indulgence in pampered appetites. Particularly is this the case in our own state. Almost exclusively an utilitarian people, our masses are too much bent upon the acquisition of wealth, to stop to enjoy it, much less to attend to laws of nature; which education and prejudice have induced them to believe are only the rules of refined luxury and effeminate fashion. This is true, not only of the lower and middle classes, but of many among the wealthy. In all their gettings they have not taken Solomon's advice and gotten wisdom. The antipodes of the man of pleasure, they forget there is a present, and live for an imaginary future. With this class a life time is not too long to devote to the acquisition of gold—but one hour in twenty-four is too much to waste upon that, which alone can enable them to enjoy it. It is ten minutes to a dinner and hours to a bargain.

Nor does this dinner, thus swallowed, not eaten, cost them a moment's thought before it is upon the table. The fowl is in the yard, the mutton in the field in the morning and upon the dish at noon. Quantity, not quality, is all they demand of their "mortels divins" (shades of Voltaire forgive us.) Les cuisiniers of the poet are as much wanted over our land as schoolmasters. Hardly less to be feared is the frying pan of old Cato, than the ignorance of the voter, a rebellion in the prima via being as much to be dreaded by the individual as a fight at the husting by the body politic.

I now proceed to the consideration of some of the most important changes produced in food by different modes of cooking, the effects of those changes upon the digestibility of various articles of our ordinary diet, and consequently upon health. Nearly all our various articles of food having an organized texture, require the agency of heat to prepare them for digestion. The exceptions to this rule are oysters and some of the fruits. Dividing, for our present purpose, the different modes of cooking into boiling, stewing, roasting, broiling, baking and frying, I will take them up as named.

Boiling is probably the most common method of preparing food for the table. If properly conducted, it is a process well calculated to render both flesh and vegetable digestible. The action of heat applied through the medium of water, the temperature of which has been raised to 212° Fahr, upon the fibrous texture of butchers' meat, fowls, or fish, is to soften the fibre, and separate the fibres from each other, and from the other tissues. "The albumen of meat being partly solid, partly liquid, the latter is coagulated by boiling water. By the united agency of water and heat, a portion of albumen, or at least a nitrogenous matter, is rendered soluble, and is, therefore, contained in the broth. The hematosin or coloring matter of the blood dissolves in, and sometimes communicates a red color to cold water, but as soon as water becomes sufficiently heated, the hematosin coagulates and forms brown floculi, which float on the top of the liquor, and constitute part of what is called the scum. The cellular tissue, the bones, the aponeuroses, and the tendons, yield, by boiling in water, gelatine. The fatty matters melt, and, except when they are contained in closed cells escaping from the meat, float on the top of the broth. The nervous or eerebral fatty matter which principally constitute the pulp of the nerves, is softened by the heat and is in part carried off during the process." During the process of boiling several products are formed by chemical reäctions yet unknown to us. We have creatine—so called by its discoverer, Chevreul—nitrogenous and crystalizable, and insoluble in alcohol. Osmazome, an extractive and highly odorous substance. Ammonia, a sulphurated compound, a volatile acid, similar to butyric acid. Boiled meat, then, consists of fibrine, coagulated albumen, gelatinous cellular tissue, fat, nervous matter and water. It has given to the water gelatine, albuminous matter, creatine, extractive matter, lactic acid, salts, and a little fatty and saccharine matter. By boiling, therefore, meat loses a portion of its nutritive properties, but at the same time it parts with some of its least digestible components. If, however, the process is properly managed, the actual loss will be trifling; for, if the meat is put into the water when it is already boiling, the albumen upon and near the surface immediately coagulates, thus closing the pores, and in a great measure preventing the escape of the juices. On the contrary, to make soup, the meat should be put in the cold water, so that gradual application of heat will dissolve the soluble matters and extract them from the insoluble fibre, tendons and bones.

Again: overboiling is injurious to some articles of food, rendering them both less digestible and less nutritious. "When gelatine," says Pereira, "is submitted to prolonged ebulition, or to a temperature exceeding 220° Fahr., it undergoes important changes. It evolves ammonia, becomes syrupy, loses its characteristic property of forming with water a jelly, and very speedily undergoes putrefaction. Thus altered, it has a disagreeable flavor, and its nutritive properties are greatly deteriorated, if not altogether destroyed. It is less digestible and readily deranges the digestive organs."—(Food and Diet.) Underboiling, of course, fails to effect the main object of the process, viz: to render the fibrous textures tender and to separate the fibres so that they may be more easily permeated by the gastric juices.

Stewing is boiling with a small quantity of water. The process is the same, but the results somewhat different; for, as stewing is usually conducted in a closely covered pan, there is little or no escape of nutritive or other principles of the articles submitted to it. The albumen and gelatine are dissolved and separated from the fibres, whilst the latter are thoroughly separated and partially broken down. A gravy is formed, containing a portion of fat and osmazome, to which it is indebted for its richness. By stewing, many wholesome and savory dishes are made, yet it is not a mode of cooking to be recommended to the dyspeptic.

Roasting, is probably, of all the various methods of preparing food, the best, both to preserve its nutritiousness and render it digestible. It is the most ancient as well as the most simple mode of cooking animal food. "The spit was probably an early cotemporary of the altar and sacrifice," says old Frederick Accum, "ere the iron age had taught men the use of metals, these roasting instruments were made of wood, and, as we find in Virgil, slender branches of the hazel tree were particularly chosen:

" ——— Statit sacer hircus ad aram,
Pinguia que in verbus torrebimus extra eolurius."

The chemical changes produced in meat by roasting are slight, but important in a culinary point of view. The watery juices, as the heat penetrates the fibrous textures, are rarified, and escape in the form of steam. The albumen coagulates. "The gelatine and the osmazome become detached from the febrine and unite with the fat, which also is liquified by the expansive properties of heat. The union of these forms a compound not to be found in the meat previously. This is retained in the interstices of the fibres, where it is formed, by the brown frothy crust, but flows abundantly from every pore where a cut is made into the meat with a knife."

Chemistry has demonstrated that roasted meat owes its peculiar odor and taste to the development of the principle called osmazome, a substance distinguished from all other constituents of animal matter, chemically, in being soluble in alcohol—and to the senses, in being extremely savory and sapid. (Accum.) "Osmazome exists in the largest quantity in the fibrous organs, or combined with fibrin in the muscles, while the tendons and other gelatinous organs, appear to be destitute of it." But a portion of the effects produced upon both meat and vegetables by roasting is entirely mechanical. The expanded juices separate the finest fibres, cellular tissue, nerves, blood-vessels, etc. The starch grains of vegetables are split and the meat turned out light and dry. But roasting, also, must be conducted fairly. If the heat is applied too quickly the meat will be scorched outside and raw within; if too slowly, the rich juices will have time to escape ere the brown crust is formed. The saddle joint, or fowl, should be neither over, nor underdone; it should be exactly done.

Broiling differs but little, chemically, from roasting. The meat being cut into small pieces, and exposed to a quick fire, the albumen of the surface is rapidly coagulated—thus preventing the escape of the juices. Hence broiled meat is extremely savory. To a delicate stomach, it is somewhat less digestible than roasted meat. Baking is roasting in a closed oven, and is objectionable on account of the empyreumatic oil which is formed by the decomposition of fat, and not allowed to make its escape. Baked meats are apt to be dry and hard, unless the operation is well conducted. Potatoes, baked, are less nutritious than boiled, as has been demonstrated by experiment on prisoners in the Glasgow Bridewell. The fact I am at a loss to explain, unless, by boiling, the starch cells are more completely broken up.

Lastly, we come to frying, the most decidedly objectionable invention of the cuisinier. "The effect of frying, upon meat, is peculiar and easily distinguished from all other modes of cookery." The meat is deprived of its own nutritious juices, in part of its gelatine, albumen, and osmazome, and then saturated with boiling fat. This boiling fat gives off carbonic acid gas, a little inflammable vapor, and an acid volatile oil, called acroleine or acroleon, whilst the fatty acids are in part set free.

I next come to consider the staff of life. Bread making—the true art of which is probably less understood than any other branch, and distressed by salt or sour masses of dough, misnamed bread, of ordinary cooking; for so often has my own stomach been shocked not to have realized this fact. Well would it be for our successors on the stage of life, if American daughters were as thoroughly instructed in this branch of housewifery as they are in less useful arts. An occasional hour might well be spared from the music stool, for the study of "Miss Leslie's Complete Cookery;" and even Euclid —now, I believe, a text-book in our female colleges—might be laid aside, with advantage, for the dough-tray and rolling-pin. The ordinary loaf, or fermented bread, is prepared from wheaten flour, salt, water, and either yeast or leaven. Bakers often add potatoes and alum. The yeast or leaven causes the sugar of the flour to undergo vinous fermentation, the result of which is the formation of carbonic acid gas and alcohol. The latter is driven out upon the application of heat; the escape of the carbonic acid being prevented by the tenacity of the dough, the latter becomes distended with gas, puffs up, assuming a vesicular texture, forming, in the language of the bake-house, the sponge. Now, if this vinous fermentation is suffered to continue over a certain definite period, the dough becomes sour, owing to the formation of acetic and lactic acids; if, on the other hand, the sponge is put into the oven too soon, fermentation is checked before the loaf is light. It is, therefore, an important point to arrest the fermentation exactly at the right moment. After baking, bread is found to weigh from 28 to 32 per cent. more than the flour used in its preparation. "In the formation of wheaten bread," says Sir H. Davey, "more than one quarter of the elements of water combine with the flour; more water is consolidated in the formation of bread from barley, and still more in that from oats; but gluten in wheat being in much larger quantity than in other grain, seems to form a combination with starch and water, whicn renders wheaten bread more digestible than other species of bread." Potatoes used in making bread assist fermentation, but containing less gluten than wheat, are less nutritious. According to Vogel, wheat bread is composed of starch, dextrine, suger-gluten, together with carbonic acid, chloride of calcium and chloride of magnesium. Leibeg states that 100 parts of fresh bread contain, on an average, 30.15 parts of carbon.

Of unfermented breads, the common biscuit need only be referred to. This is made, ordinarily, with milk and water, salt, and butter or lard. These ingredients, well mixed, form the dough, which is kneaded or beat, until a gaseous or volatile body is set free within it. This body being expanded, upon the application of heat, renders the bread light and cellular. If not well kneaded, biscuits are heavy and unfit for the table. In England, several patents have been taken out for unfermented bread. The secret of the lightness of these patented articles, depends upon the reäction between muriatic acid and carbonate of soda—chloride of sodium being formed—water and carbonic acid gas. This last product being set free, distends the dough and lightens the bread. Over fermented bread, biscuits possess several advantages. They are less troublesome to make, occupy less time, and are light and digestible, without the risk of being spoiled by carelessly timed fermentation, or bad yeast.

But I will not consume the time of the society by further details, dismissing bread, with the single remark of Dr. Paris: "All pastry is an abomination."

Having thus endeavored to give, as concisely as possible, an outline of the chemistry of cooking, or the changes produced in food by the agency of heat, I pass to the consideration of a few of the relations between these changes and digestion, and their consequent influence upon health.

Our food being cooked and placed before us, the *second* step toward digestion, is mastication. If it is an important matter to the well-being of man, that his culinary operations should be carefully conducted, according to the strictest rules of the art, it is scarcely less important, that he should give his individual attention to the agreeable task of mastication. Now, although the pleasures of the table are most ungodlike, I do not see why a necessity should not be made a rational enjoyment. I have already alluded to the steam-like rapidity with which Americans dispatch their food. Against this habit, so universal throughout the country, we, as physicians, should wage a daily, and eternal warfare. We should struggle to convince our patrons, that time is of less value than health. That teeth are not superadded, but essential organs; and that nature has not provided the human species with the gizzard of the goose, although she may occasionally misplace the head of Rome's faithful sentinels.

But to return to my subject: Thorough mastication not only minutely divides, but at the same time mixes the food with the salivary secretion,—an important adjuvant to the gastric juices. Indeed ptyeline alone possesses a certain solvent power over several proteine compounds. Mixed with saliva we have next to observe the food in the stomach, presuming it to be scientifically cooked and rationally masticated. Here for a time it undergoes no change. In the meantime the gastric juices are being poured out and are slowly penetrating the yet crude mass. Presently we can see the differences made in the kitchen. To that lucky chance for science, Beaumont's Canadian, we will give a choice bit of broiled venison, and watching through the window accident has made for us—we find that in one hour and thirty-five minutes the steak is ready to pass the pylorus. Next, we may tempt him with a slice of boiled beef—and this we find digested in two hours and forty-five minutes. Again, we give him fresh beef, fried, and three hours are required for its digestion. With eggs he is indulged in a variety of forms—raw, they digest in two hours—roasted, fifteen minutes longer is necessary; soft boiled, in three hours—and hard boiled or fried, in three hours and a half. Thus I might go on through these valuable tables of Dr. Beaumont, but they are so well known I need only refer to them.

Now, this Canadian boy, notwithstanding the misfortune which made him a martyr to science, was in good health, with unimpaired digestive powers; otherwise, I doubt not, other and widely different tables would have been made from Dr. Beaumont's experiments. If, for from twenty to thirty years, he had daily bolted fried bacon and eggs—cabbage half boiled in company with bacon—beef fried in lard, or baked crisp—fowls boiled whilst still fluttering, etc.—I feel well assured that the watches of my old friend would have been greatly prolonged. Upon how many of our patrons do you suppose like experiments could be made with like results, supposing their stomachs as accessible as was the Canadian's? But few Americans pass the prime of life without suffering from impaired digestive organs. Dyspepsia in all its varieties shortens the average of life. The nutritive properties and digestibility of much of our food is injured before it enters our stomachs. After a labored digestion it passes into the small intestines, probably in company with yet undigested portions. The mucous membrane is irritated, and cannot continue the process begun in the stomach. The chyliferous vessels refuse to absorb—the muscles of the intestines, irritated, contract—and the chyme and chyle with the crude mass—are hurried onward, and the hungry man eats, but is not satisfied. The stomach of the hardy countryman is indeed a wonderfully vigorous organ. Long may it resist serious injury from the daily attacks upon its integrity; but, after a longer or shorter time, it must complain. The victim of reckless cookery and telegraphic meals, is slow to realize that he is a diseased man. The robust farmer who can boast "I never knew a day's sickness"—who has only heard of dyspepsia as a city fashion—can hardly be persuaded that his "heart-burn" is disease, and his faithful old cook, assisted by his own habits, the cause of it.

But, gentlemen, it is our duty to teach them the truth, even to the disgrace of Cato, or the anger of ignorance.—*Southern Med. & Surg. Jour.*

